# Structural, mechanical, and electronic properties of Rh_2_B and RhB_2_: first-principles calculations

**DOI:** 10.1038/srep10500

**Published:** 2015-06-30

**Authors:** Binhua Chu, Da Li, Fubo Tian, Defang Duan, Xiaojing Sha, Yunzhou Lv, Huadi Zhang, Bingbing Liu, Tian Cui

**Affiliations:** 1 State Key Laboratory of Superhard Materials, College of physics, Jilin University, Changchun, 130012, P. R. China

## Abstract

The crystal structures of Rh_2_B and RhB_2_ at ambient pressure were explored by using the evolutionary methodology. A monoclinic *P*2_1_/*m* structure of Rh_2_B was predicted and donated as Rh_2_B-I, which is energetically much superior to the previously experimentally proposed *Pnma* structure. At the pressure of about 39 GPa, the *P*2_1_/*m* phase of Rh_2_B transforms to the *C*2/*m* phases. For RhB_2_, a new monoclinic *P*2_1_/*m* phase was predicted, named as RhB_2_-II, it has the same structure type with Rh_2_B. Rh_2_B-I and RhB_2_-II are both mechanically and dynamically stable. They are potential low compressible materials. The analysis of electronic density of states and chemical bonding indicates that the formation of strong and directional covalent B-B and Rh-B bonds in these compounds contribute greatly to their stabilities and high incompressibility.

Superhard materials have attracted considerable attention in both fundamental and technological applications due to their superior mechanical properties such as high melting temperature, high hardness, high electrical and thermal conductivity^1^. Previously, it was generally accepted that the superhard materials are those strongly covalent bonded compounds formed by light elements (B, C, N, and O), such as diamond^2^,^3^, *c*-BN^4^, BC_5_^5^, and BC_2_N^6^ etc. These superhard materials are easily to form strong three-dimensional covalent bonding networks^7^,^8^. Although diamond is the known hardest material with a measured hardness at 60–120 GPa, but it reacts easily with iron-based materials. The hardness of cubic boron nitride (*c*-BN) is second only to that of diamond. However, it can be synthesised only under high pressure and high temperature conditions which needs great cost^9^. Therefore, great efforts have been devoted to exploring novel hard and ultra-incompressible materials^10^,^11^,^12^,^13^,^14^. Recently, it was reported that partially covalent heavy transition metal (TM) boride, carbide, nitride, and oxide are found to be good candidates for superhard materials, such as ReB_2_, OsB_2_, CrB_4_ and WB_4_^15^,^16^,^17^,^18^,^19^,^20^. These reports revealed that they all possess high bulk and shear moduli. Because the compounds formed by transition metal and light elements usually possess high valence electron density and directional covalent bonds, and those covalent bonds are strong enough to improve the mechanical properties. Moreover, *d* valence electrons are considered to contribute to the hardness of transition-metal compounds. Further, these materials can be synthesized under lower pressure which leads to the low-cost synthesis condition and this is beneficial to their applications. Therefore, these pioneering studies open up a novel route for the search of novel superhard multifunctional materials.

The borides of rhodium are well known for their high melting temperature and hardness. The measured Vickers hardness of bulk RhB_1.1_ was 7-22.6 GPa, when the loads ranging from 0.49 to 9.81N^21^. Later, the 1.0 *μ*m thick RhB_1.1_ film was studied by X-ray diffraction and it possesses a hardness of 44 GPa^22^. Past studies have identified two stoichiometric compositions: RhB (hexagonal structure, No. 194, *P*6_3_/*mmc*). Wang *et al*. reported that when the pressure exceeds 22 GPa, RhB transforms from hexagonal RhB (anti-NiAs type) to the orthorhombic *Pnma* space group (FeB type)^23^, Rh_2_B (No.62, *Pnma*) has been determined that it possesses an orthorhombic structure^24^. In 1953, Richard *et al*. studied the crystal structure of Rh_2_B from X-ray rotation and Weissenberg photographs. Over the past 60 years, experimental equipment and technology have been improved dramatically, but there is no report about Rh_2_B in these years. This led us to the idea that if phase transition may occur in the Rh_2_B, which may bring about novel properties. Detailed structural, mechanical, and electronic properties theoretical investigations of Rh_2_B are also seldom. Are there compounds with higher boron contents?High boron compounds did not reveal any new phases so far. The results are worth making the effort.

In this article, we report two new phases for Rh_2_B and RhB_2_ by the first-principles calculations. Our results show that the predicted new phase of Rh_2_B belongs to the monoclinic *P*2_1_/*m* phase, which is energetically much more stable than the previously proposed *Pnma* structure in experiments. At the pressure of about 39 GPa, the *P*2_1_/*m* phase transforms to *C*2/*m* phases. While the structure type of the new phase of RhB_2_ also belong to the monoclinic *P*2_1_/*m* phase. Both of the two phases are dynamically and mechanically stable at ambient pressure. Further calculations are performed to study the properties of those high-pressure phases.

## Results and discussion

**Figure 1:**
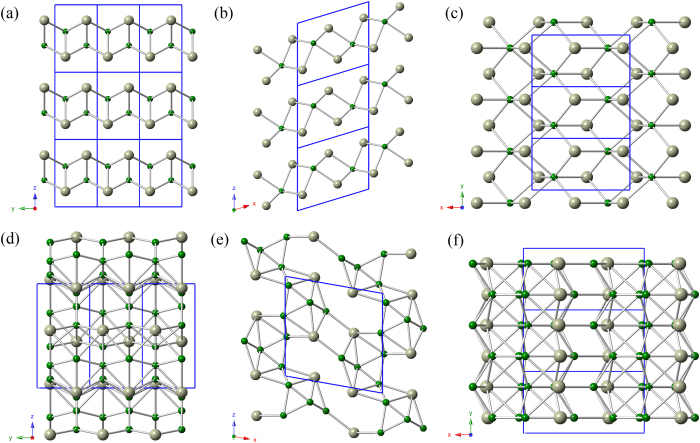
Crystal structures. The green spheres represent the B atoms, and the gray ones represent the Rh atoms. (**a**) (**b**) (**c**) Rh_2_B-I and (**d**) (**e**) (**f**) RhB_2_-II.

At ambient pressure, the variable cell simulation revealed a monoclinic Rh_2_B-I (*P*2_1_/*m*) structure as the most stable phase for Rh_2_B, as shown in Fig. 1 (a). The *P*2_1_/*m* structure contains two Rh_2_B f.u. in a unit cell (a = 5.615 Å, b = 2.873 Å, c = 4.715 Å, and β = 73.17 °), in which three inequivalent atoms Rh1, Rh2, and B occupy the Wyckoff 2*e* (0.928, 0.250, 0.769), 2*e* (0.561, 0.750, 0.230), and 2*e* (0.775, 0.250, 0.396) sites, respectively. Fig. 1 (b) along the y-axis and Fig. 1 (c) along the z-axis reveals a fundamental building block in the Rh_2_B-I structure. For RhB_2_, the predicted RhB_2_-II (*P*2_1_/*m*) is the most thermodynamically stable phase among the considered structures. Fig. 1 (d) shows the structure of RhB_2_-II. RhB_2_-II belongs to a monoclinic (*P*2_1_/*m*) space group containing two RhB_2_ f.u. in a unit cell (a = 6.044 Å, b = 3.057 Å, c = 6.116 Å and β = 100.72 °), in which Rh1, Rh2, B1, B2, B3 and B4 atoms occupy the Wyckoff 2*e* (0.306, 0.750, 0.043), 2*e* (0.292, 0.250, 0.448), 2*e* (0.015, 0.750, 0.397), 2*e* (0.963, 0.750, 0.086), 2*e* (0.582, 0.250, 0.279) and 2*e* (0.258, 0.250, 0.783) sites, respectively. From Fig. 1 (e) along the y-axis, it is seen that the boron atom sheets consist of triangle rings, within the triangle rings boron atom layer, the shortest B–B bond is 1.67 Å, which is smaller than the OsB_2_ (1.87 Å). At the same time, the boron atoms form a three dimensional space grid structure in Fig. 1 (f), and avoid the happening of the interaction between metal atoms.

**Figure 2:**
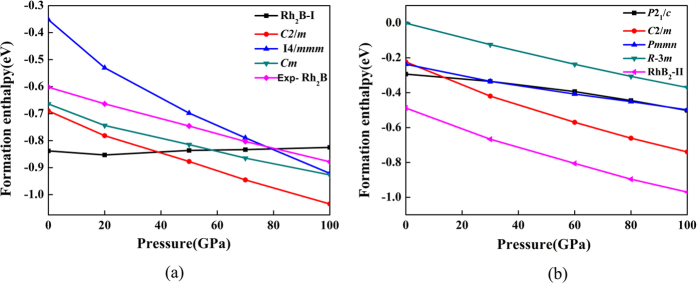
Formation enthalpy-pressure diagrams. Calculated enthalpies per unit of various structures relative to the (Rh + αB) as a function of pressure range from 0–100 GPa. (**a**) Rh_2_B-I and (**b**) RhB_2_-II

We calculated the formation enthalpy of the considered structural candidates of Rh_2_B and RhB_2_ in the pressure range of 0-100 GPa. The formation enthalpy of Rh_x_B_y_ with respect to the pure phases is investigated by the following equations Δ*H* = *H*_RhxBy_ – x*H*_Rh_ – y*H*_B_. The Rh (space group: *Fm-3m*)^25^ and *α*-Boron (space group: *R*-3*m*)^26^ were chosen as the referenced phases. Fig. 2 presents the enthalpy curves of Rh_2_B and RhB_2_ structure relative to the (Rh + αB) within the given pressure range. From Fig. 2, it can be clearly seen that the Rh_2_B-I has the lowest negative formation enthalpies at ambient pressure, and the enthalpy of Rh_2_B-I is much lower than that Rh_2_B in experiment by ∼0.16 eV per formula. This indicates that Rh_2_B-I is thermodynamically stable and can be synthesized in experiments. When the pressure is higher than 39 GPa, the phase transition from *P*2_1_/*m* phase to *C*2/m phase, because *C*2/m phase is more energetically stable in high pressure. In experiment, they used high purity rhodium metal and relatively pure boron (98.8% with slight amounts of iron and carbon)^24^. They directly mix and sinter the samples for Rh_2_B. The experimentally observed phase is a metastable phase with impurities. This can explain no presence of our predicted phases of Rh_2_B in experiments. For RhB_2_, the predicted RhB_2_-II is the most thermodynamically stable phase in our calculations, no further phase transition was observed in the high pressure range.

**Figure 3:**
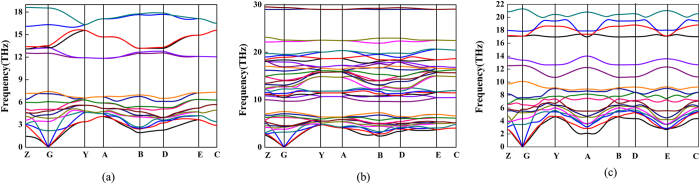
The phonon-dispersion curves. (**a**) Rh_2_B-I at 0 GPa, (**b**) RhB_2_-II at 0 GPa and (**c**) *C*2/m phase at 50 GPa respectively.

To check the dynamical stabilities of the currently predicted Rh_2_B-I and RhB_2_-II, we have calculated their phonon dispersion curves. A stable crystalline structure requires all phonon frequencies to be positive, as seen in Fig. 3 (a,b), the absence of any imaginary phonon frequency in the whole Brillouin zone for Rh_2_B-I and RhB_2_-II indicate the dynamical stabilities of the two structures at ambient pressure. In Fig. 3 (c), the calculated phonon band structure shows no soft phonon, further confirming the stability of *C*2/m phase at 50 GPa.

Elastic constants are essential for understanding the mechanical properties of a crystal. We calculated the zero-pressure elastic constants *C*_ij_ of the two phases and the elastic constants *C*_ij_ are listed in Table 1. For a stable structure, *C*_ij_ has to satisfy Born–Huangcriteria^27^: For a monoclinic crystal, the independent elastic stiffness tensor consists of thirteen components of *C*_11_, *C*_22_, *C*_33_, *C*_44_, *C*_55_, *C*_66_, *C*_12_, *C*_13_, *C*_23_, *C*_15_, *C*_25_, *C*_35_, and *C*_46_. The mechanical stability criteria is given by:


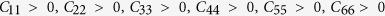



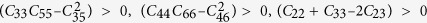



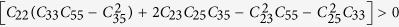



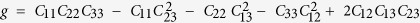



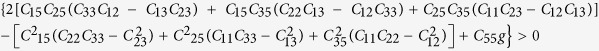


As summarized in Table 1, the Rh_2_B-I and RhB_2_-II phases satisfy all mechanical stability criteria, indicating that they are mechanical stable at ambient pressure. The calculated *C*_33_ values are bigger than that of *C*_11_ and *C*_22_ in two structures, implying that the resistance to deformation along the *c*-direction is stronger than that along the *a*-direction and *b*-direction. The calculated bulk modulus of Rh_2_B-I and RhB_2_-II is 238 and 255 GPa, respectively, both phases can be grouped into incompressible materials. Besides the bulk modulus and shear modulus, Young’s modulus could also provide a good measure of the stiffness of materials. The Young’s modulus Y is obtained by the equation: Y = (9GB)/(3B + G). Young’s modulus is defined as the ratio of stress and strain, and is used to provide a measure of the stiffness of materials in the range of elastic deformation. When the value of Y is large, the material is stiff^28^. The ratio value of *B/G* is commonly used to describe the ductility or brittleness of materials, with 1.75 as the critical value^29^. Higher (or lower) B/G value than the criteria indicates that the materials is ductile (or brittle). The *B/G* value of Rh_2_B-I is 2.74, exceeding the critical value and implying its ductile nature. RhB_2_-II also behaves in a ductile manner. The value of the Poisson’s ratio *υ* is indicative of the degree of directionality of the covalent bonds. The Poisson’s ratio *υ* is obtained by the equation: *υ* = (3B-2G)/ 2(3B + G). The typical *υ* value is 0.1 for covalent materials and 0.33 for metallic materials, respectively^30^. The Poission’s ratio of RhB_2_-II (0.277) is smaller than that of Rh_2_B-I (0.377), indicating that the directionality degree of covalent bonding of RhB_2_-II is stronger than that of Rh_2_B-I. The directionality of covalent bonding plays an important role in the hardness of materials.

**Figure 4:**
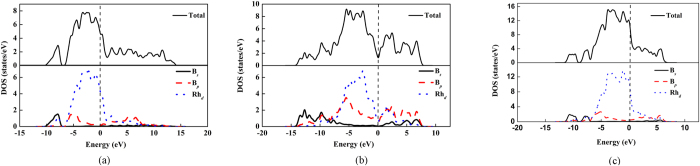
The total and partial densities of states. (**a**) Rh_2_B-I phase at 0 GPa, (**b**) RhB_2_-II phase at 0 GPa and (**c**) *C*2/m phase at 50 GPa respectively.

The electronic structure is crucial to the understanding of physical properties of materials. The electronic density of states (DOS) and the atom resolved partial density of states (PDOS) of the two phases at 0 GPa and *C*2/m phase at 50 GPa are shown in Fig. 4. In Fig. 4 (a), there is a deep valley at about −7 eV. It is a pseudogap of DOS, which is the borderline between the bonding and antibonding states. There are no clear overlap of rhodium’s d electron and boron’s p electron in range of −7 and 1 ev. In Fig. 4 (b) rhodium and boron atoms form strong covalent bonds as displayed by the much overlap of rhodium’s *d* electron and boron’s *s* electron, boron’s *p* electron curves in comparison with that of Rh_2_B-I. Indicating there is a strong covalent interaction between the B and Rh atoms in RhB_2_-II. In Fig. 4 (c), there is a deep valley at about −9 eV. There are no clear overlap of rhodium’s d electron and boron’s p electron in range of −7 and 5 ev. The finite electronic DOS at the Fermi level indicates a metallic feature for the three phases.

**Figure 5:**
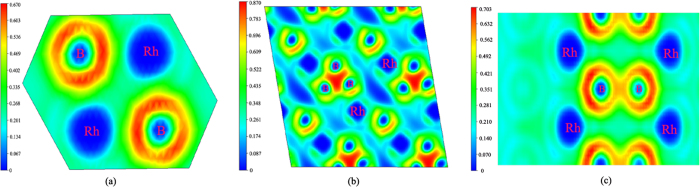
Contours of the electronic localization function (ELF). Electron localization function isosurface maps for (**a**) Rh_2_B-I at 0 GPa, (**b**) RhB_2_-II at 0 GPa, and (**c**) *C*2/m phase at 50 GPa respectively.

To gain more detailed information about the bonding character, we calculated the electronic localization function (ELF) of Rh_2_B-I , RhB_2_-II at 0 GPa and *C*2/m phase at 50 GPa. The ELF was employed to understand the electron pairing and localization of the crystal structure. It should be noted that ELF is useful in distinguishing metallic, covalent, and ionic bonds^31^. The ELF is a contour plot in real space where different contours have values ranging from 0 to 1. ELF = 1 is that there is no chance of finding two electrons with the same spin. From Fig. 5, we can see clearly that the strong bonds between Rh and B in Rh_2_B-I. This is consistent with the analysis of their DOS. The system of covalent bonds in the RhB_2_-II is significantly anisotropic, where the neighbor boron atoms form very powerful covalent bonds within the planar triangle unit, whereas Rh–B bonds are appreciably weaker. Therefore, these strong covalent bonding will increase the structural stabilities and high bulk moduli of RhB_2_-II. In Fig. 5(c) there are strong bonds between Rh and B in *C*2/m phase.

## Conclusions

In summary, we have predicted a new phase Rh_2_B-I at ambient pressure for Rh_2_B and a new phase RhB_2_-II for RhB_2_ through the *ab initio* calculation. The two new phases all belong to monoclinic *P*2_1_/*m* structure. Besides, both of the two phases are dynamically and mechanically stable at ambient pressure. Rh_2_B-I is energetically much superior to the previously proposed *Pnma* structure in the experiment. At the pressure of about 39 GPa, a phase transition occurs between the *P*2_1_/*m* and *C*2/*m* phases for Rh_2_B. For RhB_2_, RhB_2_-II is the most thermodynamically stable phase in our calculations, no further phase transition was observed in the high pressure range.

### Computation details

The evolutionary variable-cell simulations for Rh_2_B and RhB_2_ were performed at ambient pressure as implemented in the USPEX code^32^,^33^,^34^. The structure relaxation was performed by using the density functional theory implemented in the Vienna *ab initio* simulation package (VASP) code^35^,^36^,^37^. The exchange correlation functional was treated by the generalized gradient approximation (GGA)^38^ with the projector-augmented wave (PAW) potential. The tested energy cutoff 450 eV was used, the *k*-points amplings in the Brillouin zone were performed using Monkhorst-Pack method (for the hexagonal structures, a Gamma-centered *k*-points was used) to ensure that enthalpy calculations are well converged with energy differences of less than 1meV/per atom. For each candidate structure, the atomic positions, bond lengths, and cell parameters were fully optimized. Elastic constants were carried out using the CASTEP code^39^ and the bulk modulus, and shear modulus were thus estimated by using the Voigt-Reuss-Hill approximation^40^. The phonon calculations were carried out by using a supercell approach as implemented in the PHONON code^41^. The details of convergence tests have been described elsewhere^42^,^43^,^44^,^45^.

## Additional Information

**How to cite this article**: Chu, B. *et al*. Structural, mechanical, and electronic properties of Rh2B and RhB2: first-principles calculations. *Sci. Rep.* **5**, 10500; doi: 10.1038/srep10500 (2015).

## Figures and Tables

**Table 1:** Elastic Constants, Bulk Modulus (GPa), Shear Modulus (GPa), *B*/*G* ratio, Young’s modulus Υ (GPa), and Poisson’s ratio *υ* of Rh_2_B-I and RhB_2_-II at zero pressure.

	*C*_11_	*C*_22_	*C*_33_	*C*_44_	*C*_55_	*C*_66_	*C*_12_	*C*_13_	*C*_23_	*C*_15_	*C*_25_	*C*_35_	*C*_46_	*B*	*G*	*B/G*	*Y*	*υ*
Rh_2_B-I	339	350	527	56	57	73	187	143	132	-2.45	-3.39	-0.74	-10.87	238	87	2.74	232	0.337
RhB_2_-II	423	362	484	138	138	136	196	135	182	-80.74	4.28	-37.96	13.45	255	133	1.92	339	0.277
